# Long-Term Clinical Impact of Paravalvular Leak Following Transcatheter Aortic Valve Implantation

**DOI:** 10.3390/jcm14020605

**Published:** 2025-01-18

**Authors:** Cristina Aurigemma, Carlo Trani, Paola D’Errigo, Marco Barbanti, Fausto Biancari, Giuseppe Tarantini, Gian Paolo Ussia, Marco Ranucci, Gabriella Badoni, Giovanni Baglio, Stefano Rosato

**Affiliations:** 1 Department of Cardiovascular Science CUORE Fondazione Policlinico Universitario, IRCCS, A. Gemelli, 00168 Rome, Italy; cristina.aurigemma@policlinicogemelli.it; 2 Università Cattolica del Sacro Cuore, 00168 Rome, Italy; 3 National Center for Global Health, Istituto Superiore di Sanitá, 00161 Rome, Italy; paola.derrigo@iss.it (P.D.); gabriella.badoni@iss.it (G.B.); stefano.rosato@iss.it (S.R.); 4 Faculty of Medicine and Surgery, Università degli Studi di Enna “Kore”, 94100 Enna, Italy; mbarbanti83@gmail.com; 5 Department of Cardiovascular Surgery, Centro Cardiologico Monzino, IRCCS, 20138 Milan, Italy; faustobiancari@yahoo.it; 6 Division of Cardiology, Department of Cardiac, Thoracic, Vascular Sciences and Public Health, University of Padova, 35122 Padova, Italy; giuseppe.tarantini.1@unipd.it; 7 Department of Cardiology Science, Università Campus Bio-Medico, 00128 Rome, Italy; g.ussia@policlinicocampus.it; 8 Anesthesia and Cardiovascular Intensive Care Unit, IRCCS Policlinico San Donato, 20097 Milan, Italy; marco.ranucci@grupposandonato.it; 9 Italian National Agency for Regional Healthcare Services, 00187 Rome, Italy; baglio@agenas.it

**Keywords:** TAVI, TAVR, paravalvular leak, aortic stenosis

## Abstract

**Background:** Paravalvular leak (PVL) was initially recognized as one of the most common complications after transcatheter aortic valve implantation (TAVI) and has been linked to adverse clinical outcomes, including mortality. This study aims to assess the long-term clinical effects of PVL in patients undergoing TAVI with the latest generation of transcatheter aortic valves, as part of the national observational prospective multicenter study OBSERVANT II. **Methods:** OBSERVANT II included all consecutive patients with severe aortic stenosis who underwent TAVI across 28 Italian centers from December 2016 to September 2018. A total of 2125 patients were included in this analysis and stratified according to the presence of moderate-to-severe PVL (significant PVL, n = 155) versus no/trace-to-mild PVL (no significant PVL, n = 1970). The primary endpoint was 5-year major adverse cardiac and cerebrovascular events (MACCE), including all-cause death, stroke, myocardial infarction, and coronary revascularization. Five-year all cause death and re-hospitalization for heart failure (HF) were the secondary endpoints. **Results:** In our cohort, the incidence of moderate-to-severe PVL was 7%. Age, aortic anulus perimeter, and self-expandable valves were determinants of PVL. The risk of MACCE, all-cause death, and re-hospitalization for HF at the 5-year follow-up were not different between the study groups [HR = 1.07 (95% CI: 0.85–1.34) *p* = 0.571; HR = 1.10 (95% CI: 0.87–1.39) *p* = 0.435; HR = 1.20 (95% CI: 0.88–1.62) *p* = 0.245, respectively]. **Conclusions**: In this analysis of the OBSERVANT II study, moderate/severe PVL was not associated with a higher incidence of MACCE and re-hospitalization for heart failure at the 5-year follow-up.

## 1. Introduction

Nowadays, transcatheter aortic valve implantation (TAVI) is a well-established treatment option for symptomatic severe aortic stenosis (AS), particularly in the elderly and/or high-surgical-risk patients [1]. Currently, TAVI has surpassed surgical aortic valve replacement as the most common method for aortic valve replacement in developed countries, with its indications expanding to younger patients [2]. As a result, ongoing evaluation of the long-term outcomes of this minimally invasive procedure is essential. Paravalvular leak (PVL) was initially recognized as one of the most common complications after transcatheter aortic valve implantation (TAVI) and it has been linked to adverse clinical outcomes, including mortality [3,4]. In response, the latest generation of TAVI devices have been engineered with an external skirt on the aortic bioprosthesis to minimize the risk of PVL [5,6]. Previous studies have investigated the impact of this “sub-optimal” result of TAVI, reporting conflicting results [3,4,7,8,9]. Therefore, using data from the Italian national observational prospective multicenter registry, Observational Study of the Effectiveness of Transcatheter Aortic Valve Implantation with New-Generation Devices for Severe Aortic Stenosis Treatment (OBSERVANT II), we aim to report the incidence of this complication following TAVI, assess its associated risk factors, and examine its impact on long-term clinical outcomes.

## 2. Materials and Methods

### 2.1. Research Cohort

OBSERVANT II was a nationwide, prospective, multicenter observational study that involved all consecutive patients with severe aortic stenosis who received TAVI at 30 interventional cardiology centers throughout Italy from December 2016 to September 2018. For the final analysis, data were drawn from 28 centers that met the study protocol’s required minimum data quality standards [10] (the OBSERVANT II Research Group and participating centers are listed in Supplemental Material File S1). Three more centers were excluded due to insufficient completeness of computed tomography (CT) data. Ethical approval for participation in the OBSERVANT II study was obtained from the ethical committees of each participating center, and all patients provided informed consent under pseudonymization. For this sub-study, only patients who underwent TAVI using a new-generation transcatheter prostheses (Evolut R and PRO, Medtronic, Minneapolis, MN, USA; Sapien 3 Ultra, Edwards Lifesciences Corp., Irvine, CA, USA; Accurate, Boston Scientific, Marlborough, MA, USA; Lotus, Boston Scientific, Marlborough, MA, USA) via the transfemoral approach were included. Patients treated with older-generation devices, those with endocarditis, those with an emergency, and those underwent valve-in-valve or TAVI in their bicuspid valve were excluded from this analysis. Baseline characteristics, procedural information, and any adverse events that occurred during the initial hospitalization were systematically recorded in an electronic case report form for all enrolled patients [10].

### 2.2. Paravalvular Leak Assessment

Post-procedural transthoracic echocardiography (TTE) was performed before hospital discharge. Aortic regurgitation was assessed through color-flow Doppler imaging and classified based on the Valve Academic Research Consortium (VARC)-2 criteria [11], as enrollment began prior to the release of the updated VARC-3 criteria, [12] (VARC-3 criteria are largely similar). Therefore, five levels of aortic regurgitation post-TAVI were categorized: none, trivial, mild, moderate, or severe. The assessment of both native and post-TAVI aortic regurgitations was performed according to the guidelines of the European Association of Echocardiography [13] and the recommendations of the American Society of Echocardiography [14], utilizing a multiparametric and integrative approach rather than relying on a single measurement. In cases of post-TAVI aortic regurgitation, which are often paravalvular, the evaluation placed greater emphasis on the circumferential extent of the paravalvular jets, as assessed just below the bioprosthesis in the short-axis view, rather than on other parameters. The circumferential extent refers to the proportion of the prosthetic valve’s circumference that is affected by the regurgitant jet. In the short-axis view, the length of the jet along the valve’s curvature is measured and compared to the total perimeter of the valve ring. This measurement is expressed as a percentage, providing a quantitative assessment of the PVL’s severity. For instance, a circumferential extent of less than 10% is generally indicative of mild PVR, 10–30% suggests moderate PVL, and more than 30% is associated with severe PVL [15]. Assessing the circumferential extent of paravalvular jets in the short-axis view is considered to be a key component in evaluating post-TAVI aortic regurgitation, providing valuable information on the severity of PVR.

### 2.3. Clinical Follow-Up and Outcomes

Adverse events occurring after hospital discharge were monitored by linking data with the National Hospital Discharge Records database from the Italian Ministry of Health, along with other administrative databases accessible through a partnership with the Italian National Program for Outcome Evaluation (PNE-AGENAS). This linkage ensured a complete 5-year follow-up for all enrolled patients connected to the administrative databases. The primary endpoint of the study was major adverse cardiac and cerebrovascular events (MACCE), a composite endpoint that includes all-cause mortality, rehospitalizations for non-fatal myocardial infarction (MI), non-fatal stroke, and coronary revascularizations (CABG, PCI) within 5 years. All-cause mortality alone was included as a secondary endpoint to differentiate the impact of fatal versus non-fatal events, while rehospitalization for heart failure served as another secondary endpoint to highlight the hemodynamic consequences of PVL.

### 2.4. Statistical Analysis

Covariates and outcomes were reported as counts and percentages for categorical variables, and mean and standard deviation for continuous variables. The Mann–Whitney U, Kruskal–Wallis, and chi-square tests were used to compare baseline and operative covariates between the two study groups. Fisher’s exact test was used when cells had expected frequencies of <5. As the study groups had a heterogeneous distribution of baseline and operative covariates, a logistic regression with backward selection was performed to identify the independent predictors of PVL. Unadjusted differences in 5-year MACCE and mortality were evaluated using the Kaplan–Meier method with the log-rank test, while unadjusted differences in the in the 5-year rehospitalization for HF was evaluated using a competing risk approach (death as a competing risk) and Gray’s test. Multivariate Cox semi-parametric regression models for MACCE and all-cause mortality were implemented to investigate the differences between the study groups, adjusted for the main baseline and operative risk factors. A Fine and Gray model was used for the competing risk analysis of time to rehospitalization for HF, with death as the competing risk. A stepwise approach was used to automatically select variables associated with analyzed outcomes using the following covariates: female sex, age, previous MI, diabetes, peripheral artery disease, chronic obstructive pulmonary disease, oxygen therapy, dialysis, pulmonary hypertension, active cancer, neurological disfunction, liver diseases, previous cardiac surgery, previous percutaneous coronary intervention, coronary artery disease, frailty class, EuroSCORE II, NYHA class, BMI class, eGFR class, moderate/severe mitral regurgitation, left ventricular ejection fraction, aortic mean gradient, self-expandable transcatheter valve, valve post-dilation, and anulus perimeter. Statistical analysis was performed using the SAS statistical package v. 9.4 (SAS Institute Inc., Cary, NC, USA).

## 3. Results

**Figure 1:**
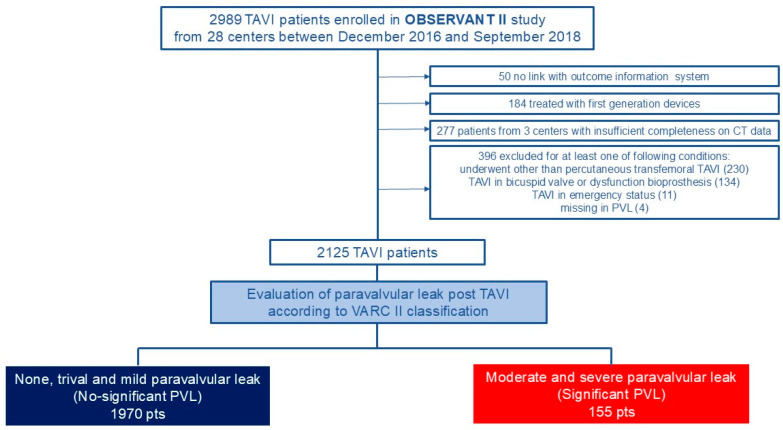
Study flow chart.

The OBSERVANT II registry enrolled 2989 patients with severe aortic stenosis who underwent TAVI between December 2016 and September 2018 in 28 centers across Italy. After exclusion criteria were applied, a cohort of 2125 patients constituted the study population for this analysis. Based on the PVL assessment, the enrolled population was divided into two groups: 1970 patients with no, trivial, or mild PVL (no significant PVL) and 155 (7%) with moderate or severe PVL (significant PVL) (Figure 1). The incidence of severe PVL was 0.3%.

The baseline characteristics of the patients included in the study are presented in Table 1, which highlights some differences between the two groups. Patients with no significant PVL were younger compared to patients with significant PVL (82.1 ± 6.2 vs. 83.4 ± 6, *p* = 0.012). Diabetes was more frequent in the group with no significant PVL compared to those with significant PVL (28.9% vs. 21.3%, *p* = 0.044). Instead, peripheral artery disease and previous percutaneous coronary intervention were more frequent in the group with significant PVL (23.4% vs. 14.3%, *p* = 0.002 and 18.7% vs. 12.4%, *p* = 0.024, respectively). The EuroSCORE II was not different between the two groups. Table 2 summarizes the main echocardiography, anatomical, and procedural features. The incidence of associated mitral regurgitation and the left ventricle ejection fraction was no different between the two groups. Instead, the mean aortic gradient was higher in patients with significant PVL compared to patients with no significant PVL (49.8 ± 15.3 mmHg vs. 47.3 ± 14.7 mmHg, *p* = 0.048). The aortic anulus perimeter and was bigger in patients with significant PVL (76.2 ± 0.9 mm vs. 74.4 ± 0.9 mm, *p* = 0.019). The use of a self-expandable valve was more frequent in patients with significant PVL (28.5% vs. 8.4%, *p* < 0.001). As expected, post-dilation was performed more frequently in patients with significant PVL (41.9% vs. 22.1%, *p* < 0.001). Valve migration also occurred more frequently in the group with significant PVL (2.6% vs. 0.01%, *p* = 0.001). Age, aortic anulus perimeter, and a self-expandable valve were predictors of significant PVL (OR 1.04, 95% CI 1.01–1.08, *p* = 0.018; OR 1.41, 95% CI 1.09–1.83, *p* = 0.010; OR 3.44, 95% CI 1.75–6.67, *p* < 0.001, respectively) (Table 3).

### Clinical Outcomes

**Figure 2:**
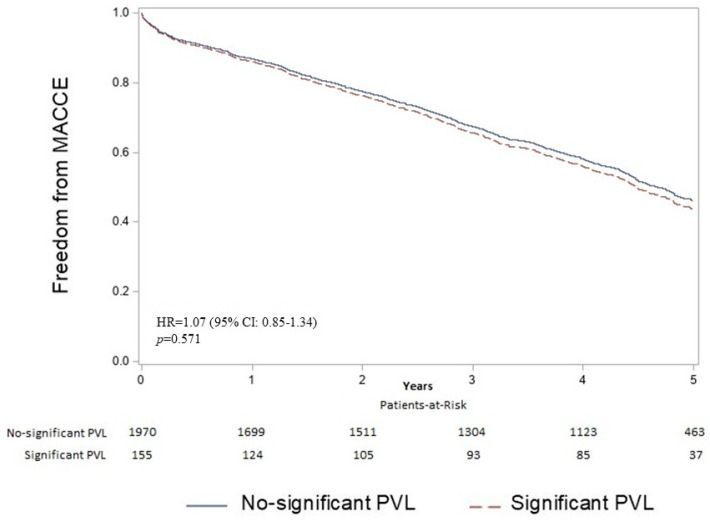
Adjusted survival curves for freedom from MACCE. The figure shows the adjusted survival curves for freedom from MACCE (all-cause death, non-fatal MI, non-fatal stroke, CABG, and PCI) in the study population stratified according to paravalvular leak (PVL). No-significant PVL included no, trivial, or mild paravalvular leak. Significant PVL included moderate or severe paravalvular leak. Table 4 reported the factors used in the multivariate model.

**Figure 3:**
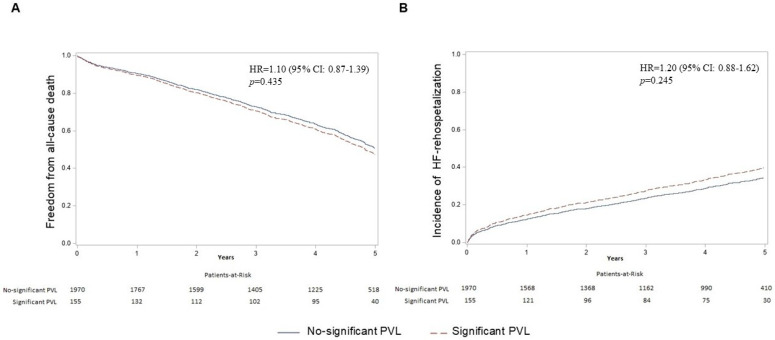
Death and incidence of heart failure rehospitalization according to the severity of paravalvular leak. The figure shows the adjusted survival curves from all-cause death (A) and the adjusted incidence curves for heart failure rehospitalization (B) stratified according to paravalvular leak (PVL). No significant PVL was defined as no, trivial, or mild paravalvular leak. Significant PVL was defined as moderate or severe paravalvular leak. Table 4 reported the factors used in the multivariate model.

The mean follow-up period was 3.7 years (4.8 years for patients who were alive at the end of follow-up). The unadjusted analysis for the considered outcomes is reported in the Supplementary Materials (Figures S1–S3). The adjusted survival curves free from MACCE are reported in Figure 2 and showed no difference between patients with significant PVL and no significant PVL (HR= 1.07, 95% CI 0.85–1.34, *p* = 0.571). Furthermore, the rate of HF rehospitalization and the incidence of 5-year all-cause death were also no different between the two groups (HR= 1.20, 95% CI 0.88–1.62, *p* = 0.245; HR= 1.10, 95% CI 0.87–1.39, *p* = 0.435, respectively, Figure 3). In Table 4, the multivariate Cox regression models for each of the considered outcomes are reported. In the analyzed cohort, a moderate/severe level of PVL compared with none/mild level was not an independent predictor of MACCE (HR 1.07, 95% CI 0.85–1.34, *p* = 0.517), all-cause mortality (HR 1.10, 95% CI 0.87–1.39, *p* = 0.435), and HF-rehospitalization (HR 1.20, 95% CI 0.88–1.62, *p* = 0.245) at the 5-year follow-up. Instead, age, comorbidities, and hemodynamic parameters influenced long-term outcomes after TAVI. In particular, each year of age increased the hazard of MACCE (HR 1.02, 95% CI 1.01–1.03, *p* = 0.002) and of all-cause mortality (HR 1.02, 95% CI 1.01–1.04, *p* < 0.001). Moreover, comorbidities such as pulmonary hypertension (HR for MACCE 1.50, 95% CI 1.16–1.93, *p* = 0.002; HR for death 1.65, 95% CI 1.28–2.14, *p* < 0.001; HR for HF rehospitalization 1.63, 95% CI 119–2.23, *p* = 0.003) and impaired renal function (eGFR <30 vs. >60: HR for MACCE 1.76, 95% CI 1.43–2.17, *p* < 0.001; eGFR <30 vs. >60: HR for death 1.87, 95% CI 1.51–2.32, *p* < 0.001; eGFR <30 vs. >60: HR for HF rehospitalization 1.83, 95% CI 1.39–2.39, *p* < 0.001) were among the most important predictors for all of the considered outcomes.

## 4. Discussion

Transcatheter aortic valve implantation (TAVI) was initially developed as an alternative treatment for severe symptomatic aortic valve stenosis in patients with a high or prohibitive surgical risk. Nowadays, the indication for TAVI is expanding to younger and lower-risk patient groups [5,6]. Therefore, it is essential to consistently evaluate the long-term outcomes of this percutaneous treatment for aortic stenosis. In this sub-analysis of the OBSERVANT II study, we observed the following:

- The incidence of moderate/severe PVL after TAVI was 7%, with 0.3% classified as severe.
- Major predictors of moderate/severe PVL were age, aortic annulus perimeter, and the use of self-expandable valve devices.
- Moderate/severe PVL after TAVI had no clinical impact at the 5-year follow-up.

The incidence of PVL was initially quite common, with up to 22% of patients experiencing a moderate-to-severe paravalvular leak [7]. Although TAVI technologies have advanced and operator experience has improved, the rate of moderate-to-severe PVL remains higher after TAVI compared to SAVR [15,16]. In our sub-analysis, we observed an incidence of moderate/severe PVL of 7% in TAVI procedures performed at 25 Italian centers between December 2016 and September 2018. Our findings show a lower incidence of moderate/severe PVL compared to recent data from the France-TAVI registry, which reported an incidence of 19.4% between 2010 and 2019 [7].

Consistent with previous studies, our sub-analysis also identified self-expandable valves and larger annulus dimensions as predictors of PVL [17,18,19]. Additionally, age emerged as a predictor of PVL in our analysis. This finding could be attributed to increased valvular calcification in older patients. However, we unfortunately lack data on aortic annulus and leaflet calcifications, which are important determinants of PVL, as highlighted in previous studies. Another contributing factor might be the acceptance of suboptimal outcomes (such as lower rates of post-dilatation to optimize procedural results) in elderly patients, aimed at minimizing potential procedure-related complications.

The higher prevalence of post-dilation in cases with significant PVL underscores the importance of procedural optimization. Advanced imaging techniques, such as pre-procedural computed tomography, alongside precise valve positioning and deployment strategies, are essential for minimizing PVL. These techniques help to improve annular sizing, optimize prosthesis expansion, and ensure appropriate sealing, thereby reducing the need for corrective interventions such as post-dilation.

The clinical impact of a moderate/severe paravalvular leak remains debated. Previous studies have indicated that a mild PVL is frequently observed following TAVI and is generally associated with a favorable prognosis [20]. However, other research has demonstrated that patients with a PVL experience higher long-term mortality rates [21,22]. A meta-analysis of 38 observational studies, including over 25,000 patients, found that any degree of PVL post-TAVI negatively impacts both overall survival and functional status [23]. Recent data from the France-TAVI registry confirmed the negative impact of moderate/severe PVL on clinical outcomes, reporting on 20,878 patients who underwent TAVI between 2010 and 2019 at a 6.5-year follow-up [7]. On the other hand, data from the Polish national POL-TAVI registry confirmed that PVL remains a frequent problem after TAVI but demonstrated no differences in six-month mortality rates between patients with no or mild PVL compared to those with moderate PVL [9]. Similarly, in our sub-analysis, we found no differences in the incidence of MACCE or HF rehospitalization between patients with no significant PVL and those with significant PVL at long-term follow-up.

The low prevalence of significant PVL in our cohort may have limited the ability to detect differences in MACCE outcomes, especially over a long follow-up period. Advances in patient selection, procedural techniques, and device design may have reduced PVL’s clinical impact. Additionally, factors like age, comorbidities, and hemodynamic conditions were stronger predictors of MACCE, likely having a greater influence on long-term outcomes than PVL alone. These findings highlight the complexity of PVL’s clinical impact and suggest the need for further research on its relationship with comorbidities and long-term outcomes.

### Limitations

This was an observational study, and like any observational analysis, it carries inherent limitations, including potential residual confounding from both measured and unmeasured factors. However, the study benefits from being large scale, encompassing all commercial TAVI procedures performed in Italy within a recent period, and from being linked to national registries, which ensure comprehensive long-term follow-up for all patients. Additionally, there may be some degree of underreporting or missing data. Lastly, the association between aortic root dimensions, calcification, and PVL remains unexplained. Aortic annulus and leaflet calcifications are important predictors of PVL after TAVI. However, data regarding the extent and distribution of calcifications were not collected in this registry, limiting the ability to assess their role as predictors of PVL in this study.

## 5. Conclusions

In conclusion, our analysis from the OBSERVANT II registry revealed that the incidence of significant PVL (moderate/severe) after TAVI procedures was 7%. Various risk factors, primarily linked to patient anatomy and TAVI device selection, have been identified for this complication. Careful pre-procedural screening for factors such as annulus size, valve morphology, and the extent of calcification of the aortic annulus and leaflets is recommended to minimize the risk of significant PVL. Advanced imaging modalities, including computed tomography angiography (CTA), play a critical role in guiding appropriate prosthesis selection and sizing. Procedural strategies, such as optimal valve positioning and post-dilatation, when necessary, are crucial in reducing the incidence of significant PVL. Incorporating these approaches into clinical practice may improve the procedural outcomes of a TAVI procedure. Significant PVL was not associated with an increased risk of MACCE, all-cause death, or HF rehospitalization at the 5-year follow-up.

## Figures and Tables

**Table 1:** Baseline characteristics of study population.

	No Significant PVL (1970)	Significant PVL (155)	*p*-Value
Female Sex n (%)	1117 (56.7)	85 (54.8)	0.653
Age	82.1 ± 6.2	83.4 ± 6.1	0.012
Previous MI, n (%)	41 (2.1)	6 (3.9)	0.320
Diabetes, n (%)	566 (28.9)	33 (21.3)	0.044
PAD, n (%)	279 (14.3)	36 (23.4)	0.002
COPD, n (%)	300 (15.2)	24 (15.5)	0.936
Oxygen therapy, n (%)	66 (3.4%)	3 (1.9%)	0.336
Dialysis, n (%)	43 (2.2)	5 (3.2)	0.406
Pulmonary hypertension, n (%)	105 (5.3)	9 (5.8)	0.801
Active cancer, n (%)	76 (3.9)	5 (3.2)	0.666
Neurological disfunction, n (%)	45 (2.3%)	6 (3.87%)	0.214
Liver disease, n (%)	30 (1.5%)	3 (1.9%)	0.696
Previous cardiac surgery, n (%)	232 (11.8)	15 (9.7)	0.432
Coronary artery disease, n (%)			0.014
1 vessel	280 (14.3)	24 (15.7)	
2 or more vessels	173 (8.9)	24 (15.7)	
Previous CABG, n (%)	180 (9.1)	11 (7.1)	0.392
Previous PCI, n (%)	244 (12.4)	29 (18.7)	0.024
GSS frailty 3–4, n (%)	396 (20.1)	32 (20.6)	0.871
EuroSCORE II	6.5 ± 6.4	7.4 ± 7.0	0.082
NYHA III/IV, n (%)	1407 (72.1)	117 (75.5)	0.362
BMI class, n (%)			<0.001
≤25	821 (41.8)	87 (56.1)	
25–30	708 (36.1)	52 (33.5)	
>30	433 (22.1)	16 (10.3)	
eGFR class, n (%)			0.261
<30	198 (10.1)	22 (14.2)	
30–60	870 (44.2)	64 (41.3)	
>60	899 (45.7)	69 (44.5)	

PAD, peripheral artery disease; COPD, chronic obstructive pulmonary disease; CABG, coronary artery bypass grafting; PCI, percutaneous coronary intervention; GSS, geriatric status scale; NYHA, New York Heart Association; BMI, body mass index; eGFR, estimated glomerular filtration rate.

**Table 2:** Echocardiography, anatomical, and procedural characteristics of study population.

	PVL None/Mild (1970)	PVL Moderate/Severe (155)	*p*-Value
***Echocardiography Features***			
Mitral regurgitation, moderate/severe, n (%)	639 (32.5)	55 (35.71)	0.416
LVEF	53.9 ± 11.1	53.4 ± 11.3	0.370
Aortic mean gradient	47.3 ± 14.7	49.8 ± 15.3	0.048
***Anatomical Features***			
Aortic valve area	4.39 ± 1.0	4.5 ± 1.1	0.220
Aortic valve perimeter	74.4 ± 8.6	76.2 ± 9	0.019
Aortic anulus diameter	22.4 ± 2.3	23.1 ± 2.8	0.039
***Procedural Features***			
SelfExpTAVI, n (%)	1410 (71.5)	13 (91.6)	<0.001
Valve re-sheathing, n (%)	366 (18.6)	28 (18.1)	0.641
Valve migration, n (%)	8 (0.4)	4 (2.6)	0.001
Valve post-dilatation, n (%)	435 (22.1)	65 (41.9)	<0.001
Valve pre-dilatation, n (%)	900 (45.8)	72 (46.5)	0.871

LVEF, left ventricular ejection fraction.

**Table 3:** Determinants of moderate-to-severe paravalvular leak after TAVI.

	OR	95% CI	*p* Value
Age	1.04	1.01–1.08	0.018
Female sex	1.15	0.74–1.77	0.532
Anulus perimeter	1.41	1.09–1.83	0.010
Self-expandable transcatheter valve	3.44	1.75–6.67	<0.001
Mean gradient	1.01	1.00–1.02	0.138
Valve post-dilatation	2.02	1.38–2.96	<0.001

**Table 4:** Results of multivariable analysis for 5-year MACCE, all cause death, and rehospitalization for HF.

		MACCE		All-Cause Death		HF Rehospitalization	
		HR (95% CI)	*p*-Value	HR (95% CI)	*p*-Value	HR (95% CI)	*p*-Value
Age		1.02 (1.01–1.03)	0.002	1.02 (1.01–1.04)	<0.001	1.00 (0.99–1.02)	0.789
Female sex		0.72 (0.63–0.81)	<0.001	0.71 (0.62–0.81)	<0.001	0.96 (0.81–1.14)	0.627
Significant PVL		1.07 (0.85–1.34)	0.571	1.10 (0.87–1.39)	0.435	1.20 (0.88–1.62)	0.245
Aortic mean gradient		0.99 (0.99–1.00)	0.001	0.99 (0.99–1.00)	<0.001	0.98 (0.97–0.98)	<0.001
Previous MI		1.39 (0.96–2.01)	0.084	1.30 (0.88–1.92)	0.185	-	
Oxygen Therapy		1.38 (1.00–1.89)	0.049	1.38 (0.99–1.91)	0.056	1.62 (1.07–2.45)	0.023
Active cancer		-	-	-	-	0.61 (0.34–1.09)	0.096
Pulmonary Hypertension		1.50 (1.16–1.93)	0.002	1.65 (1.28–2.14)	<0.001	1.63 (1.19–2.23)	0.003
PAD		1.19 (1.01–1.40)	0.042	1.22 (1.03–1.44)	0.025	-	
COPD		1.29 (1.09–1.53)	0.004	1.42 (1.19–1.69)	<0.001	1.58 (1.26–1.97)	<0.001
Diabetes		1.36 (1.19–1.56)	<0.001	1.38 (1.20–1.58)	<0.001	1.47 (1.24–1.75)	<0.001
Dialysis		1.63 (1.14–2.33)	0.007	1.66 (1.16–2.39)	0.006	-	
Neurological dysfunction		1.36 (0.95–1.96)	0.097	-	-	-	
Liver disease		1.48 (0.90–2.42)	0.120	1.88 (1.15–3.09)	0.012	-	
Class NYHA II/III		1.18 (1.02–1.36)	0.025	1.19 (1.02–1.38)	0.025	1.26 (1.04–1.53)	0.019
GSS Frailty 3–4 vs. 1–2		1.19 (1.02–1.38)	0.024	1.23 (1.05–1.44)	0.009	-	
CAD	1 vs. 0 vessels	-		-		0.82 (0.64–1.06)	0.126
	2 or more vs. 0 vessels	-		-		1.07 (0.82–1.41)	0.603
eGFR	30–60 vs. 60+	1.09 (0.95–1.25)	0.225	1.10 (0.96–1.27)	0.184	1.04 (0.86–1.25)	0.689
	0–30 vs. 60+	1.76 (1.43–2.17)	<0.001	1.87 (1.51–2.32)	<0.001	1.83 (1.39–2.39)	<0.001
BMI	<25 vs. 25–30	1.22 (1.06–1.40)	0.006	1.34 (1.16–1.55)	<0.001	-	
	≥30 vs. 25–30	1.00 (0.84–1.20)	0.999	1.07 (0.89–1.29)	0.490	-	
Mitral regurgitation, moderate/severe		1.10 (0.97–1.26)	0.151	1.13 (0.99–1.30)	0.081	1.27 (1.07–1.51)	0.007
EuroSCORE II		-		-	-	1.01 (1.00–1.02)	0.189

PAD, peripheral artery disease; COPD, chronic obstructive pulmonary disease; NYHA, New York Heart Association; GSS, geriatric status scale; CAD, coronary artery disease; BMI, body mass index; eGFR, estimated glomerular filtration rate.

## Data Availability

The raw data supporting the conclusions of this article will be made available by the authors on request.

## Supplementary Materials

The following supporting information can be downloaded at: https://www.mdpi.com/article/10.3390/jcm14020605/s1, File S1. Observant II Research Group and Participating Centers. Figure S1. Unadjusted survival curves from MACCE. Figure S2. Unadjusted survival curves from all causes of death. Figure S3. Unadjusted survival curves from heart failure rehospitalization.
