# Supplementary material for: Long-Term Clinical Impact of Paravalvular Leak Following Transcatheter Aortic Valve Implantation
Source: J Clin Med. 2025 Jan 18;14(2):605. doi: 10.3390/jcm14020605 (PMC11765977; doi:10.3390/jcm14020605)
Supplement: Supplementary file 1 [file jcm-14-00605-s001.zip › jcm-3387878-supplementary.pdf]

SUPPLEMENTAL MATERIAL

Supplemental File S1

OBSERVANT II RESEARCH GROUP

Coordination

Fulvia Seccareccia, Paola D'Errigo, Stefano Rosato, Gabriella Badoni.

National Centre for Global Health - Istituto Superiore di Sanità, Rome, Italy;

Collaborators for the "Ricerca Finalizzata 2016" (PE-2016-02364619)

Corrado Tamburino (PI), Davide Capodanno (Co-PI), Marco Barbanti. A.O.U. Policlinico "G. Rodolico – San Marco" – University of Catania, Catania, Italy

Fausto Biancari. Helsinki University Hospital and University of Helsinki, Helsinki, Finland; Oulu University Hospital, Oulu, Finland

Giovanni Baglio, Francesco Cerza. Agenzia Nazionale per i Servizi Sanitari Regionali (Age.Na.S) – PNE, Rome, Italy

Andrea Marcellusi. Faculty of Economics, University of Rome "Tor Vergata", Rome, Italy

Representatives of the Scientific Societies

- IFC - Italian Federation of Cardiology

Gennaro Santoro. Fondazione "G. Monasterio" CNR/Tuscany Region for the Medical Research and Public Health, Massa, Italy

Gian Paolo Ussia. Campus Bio-Medico University of Rome, Rome, Italy

- GISE – Italian Society of Interventional Cardiology

Giuseppe Musumeci. S. Croce e Carle Hospital, Cuneo

Francesco Bedogni. IRCCS Policlinico S. Donato, S. Donato Milanese, Milan, Italy

Sergio Berti. Fondazione "G. Monasterio" CNR/Tuscany Region for the Medical Research and Public Health, Massa, Italy

Giuseppe Tarantini. University of Padova, Padova, Italy

- ITACTA - Italian Association of Cardiothoracic Anesthesia

Massimo Baiocchi. Policlinico Sant'Orsola, Bologna, Italy

Marco Ranucci. IRCCS Policlinico S. Donato, S. Donato Milanese, Milan, Italy

Institutional collaborations

- National

Domenico Mantoan. Agenzia Nazionale per i Servizi Sanitari Regionali (Age.Na.S), Rome, Italy

- Italian Regional Authorities

Rossana De Palma. Emilia Romagna Region

Salvatore Scondotto. Sicily Region

Anna Orlando. Piemonte Region

OBSERVANT II PARTICIPING CENTERS

1. A.O.U. Città della Salute e della Scienza di Torino (TO) - Mauro Rinaldi, Stefano Salizzoni
2. A.O. S. Croce e Carle (CN) - Giuseppe Musumeci, Giorgio Baralis
3. A.O. SS. Antonio e Biagio e Cesare Arrigo (AL) - Gianfranco Pistis, Maurizio Reale
4. I.R.C.C.S Policlinico San Donato (San Donato Milanese - MI) - Francesco Bedogni, Giovanni Bianchi
5. I.R.C.C.S Multimedica (Sesto San Giovanni - MI) - Flavio Airoldi, Iassen Michev
6. Fondazione I.R.C.C.S. Policlinico San Matteo (PV) - Maurizio Ferrario, Umberto Canosi
7. ASST Lecco - Ospedale "A. Manzoni" (LC) - Luigi Piatti, Gianluca Tiberti
8. ASST degli Spedali Civili - Presidio Ospedaliero di Brescia (BS) - Federica Ettori (retired), Salvatore Curello, Marianna Adamo
9. I.R.C.C.S Ospedale San Raffaele (MI) - Antonio Colombo, Matteo Montorfano, Marco Ancona,
10. ASST Monza & Brianza - Ospedale S. Gerardo (MB) - Virgilio Colombo, Ivan Calchera
11. Fondazione Poliambulanza (BS) - Ornella Leonzi, Diego Maffeo
12. ASST Papa Giovanni XXIII (BG) - Orazio Valsecchi, Federica Roncali, Angelina Vassileva
13. Policlinico di Monza (MB) - Filippo Scalise, Giovanni Sorropago
14. A.O. di Padova - Centro Gallucci (PD) - Giuseppe Tarantini, Alessandro Schiavo
15. Hesperia Hospital (MO) - Giuseppe D'Anniballe, Davide Gabbieri
16. A.O.U. di Parma (PR) - Luigi Vignali, Michela Bollettino
17. A.O.U. Careggi (FI) - Carlo Di Mario, Francesco Meucci
18. A.O.U. Senese - Ospedale Santa Maria alle Scotte (SI) - Carlo Pierli (retired), Massimo Fineschi, Alessandro Iadanza
19. Fondazione Toscana Gabriele Monasterio - Ospedale del Cuore "G. Pasquinucci" (MS) - Sergio Berti, Giuseppa Lo Surdo

20. Ospedale San Filippo Neri (RM) - Giulio Speciale, Andrea Bisciglia

21. Fondazione Policlinico Universitario Agostino Gemelli IRCCS - Università Cattolica del Sacro Cuore (RM) - Carlo Trani, Diana Verdirosi

22. A.O. San Camillo Forlanini (RM) - Roberto Violini, Laura Zappavigna

23. A.O. San Giuseppe Moscati (AV) - Emilio Di Lorenzo, Michele Capasso

24. A.O.U. Federico II (NA)- Giovanni Esposito, Fabio Magliulo

25. A.O.U. OO.RR. San Giovanni di Dio e Ruggi d'Aragona (SA) - Pietro Giudice, Tiziana Attisano

26. A.O.U.C. Policlinico di Bari (BA) - Alessandro Santo Bortone, Emanuela De Cillis

27. A.O.U. Policlinico-Vittorio Emanuele, Università di Catania (CT) - Corrado Tamburino, Marco Barbanti

28. Centro Cuore Morgagni - Pedara (CT) - Sebastiano Immè, Martina Patanè

Figure S1

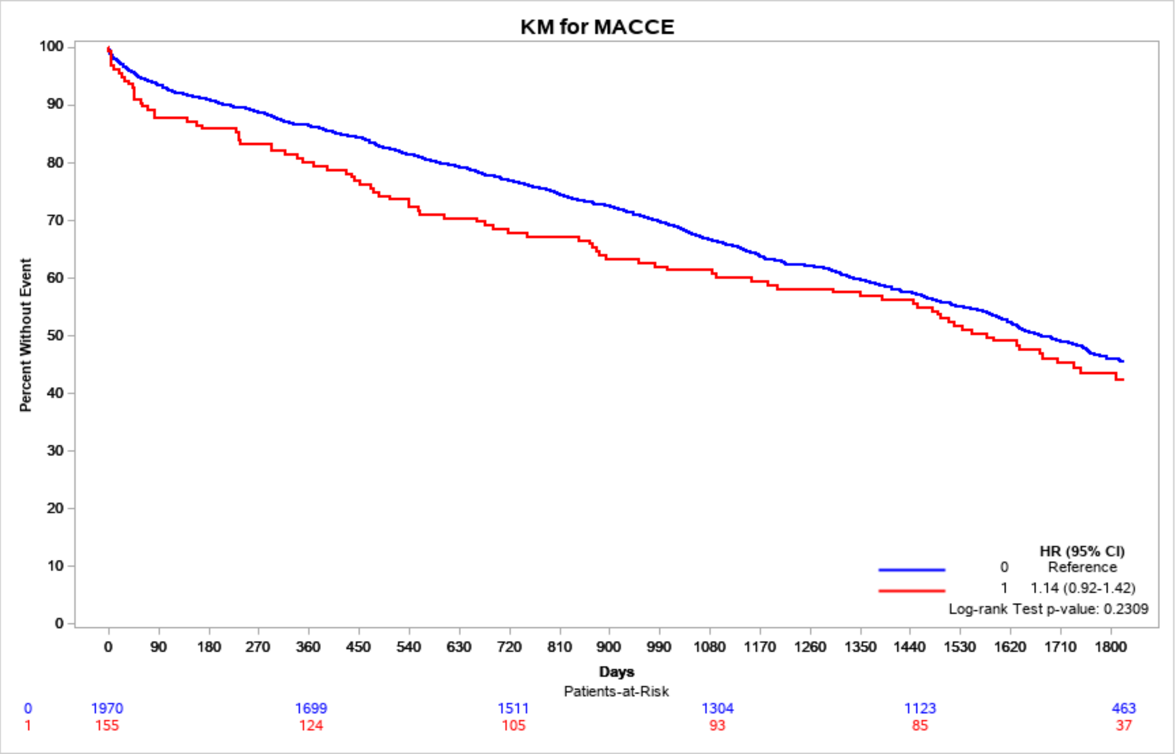

The figure shows the unadjusted survival curves from MACCE (all death, non-fatal MI, non-fatal stroke CABG and PCI). 0 (No-significant PVL: none, trivial and mild paravalvular leak) 1 (Significant PVL: moderate and severe paravalvular leak).

Figure S2

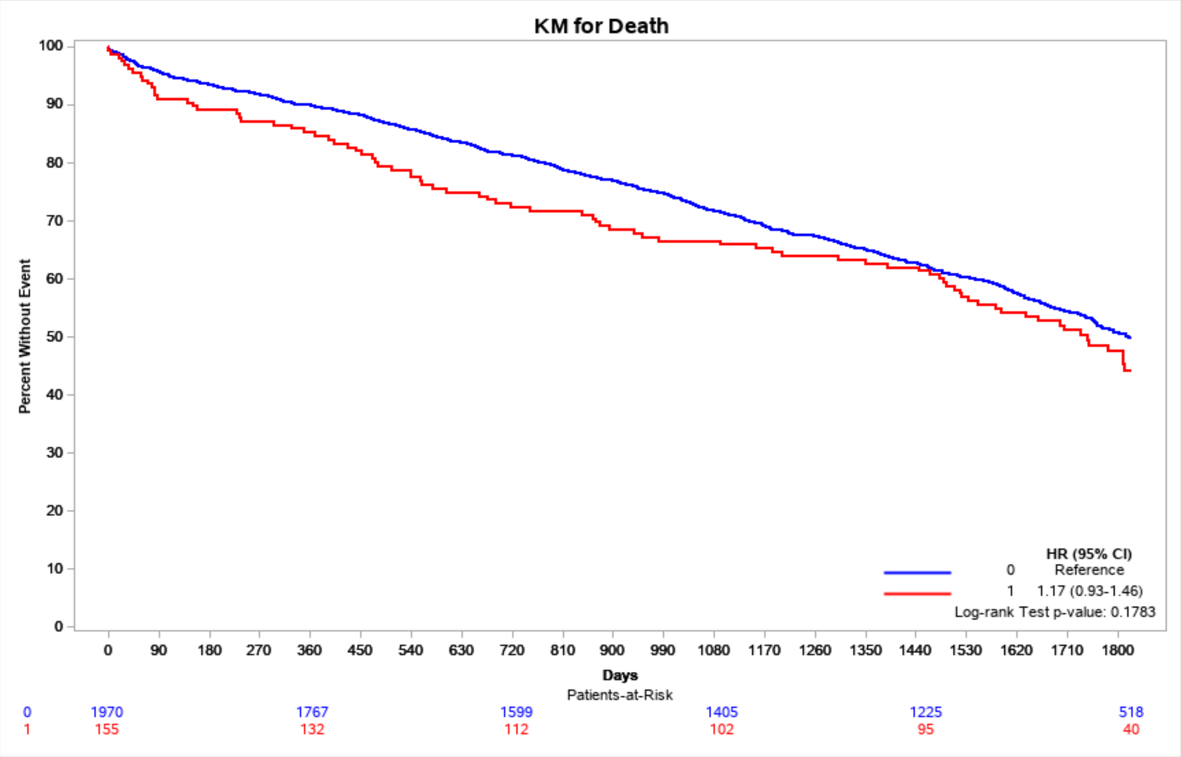

The figure shows the unadjusted survival curves from all cause of death. 0 (No-significant PVL: none, trivial and mild paravalvular leak) 1 (Significant PVL: moderate and severe paravalvular leak).

Figure S3

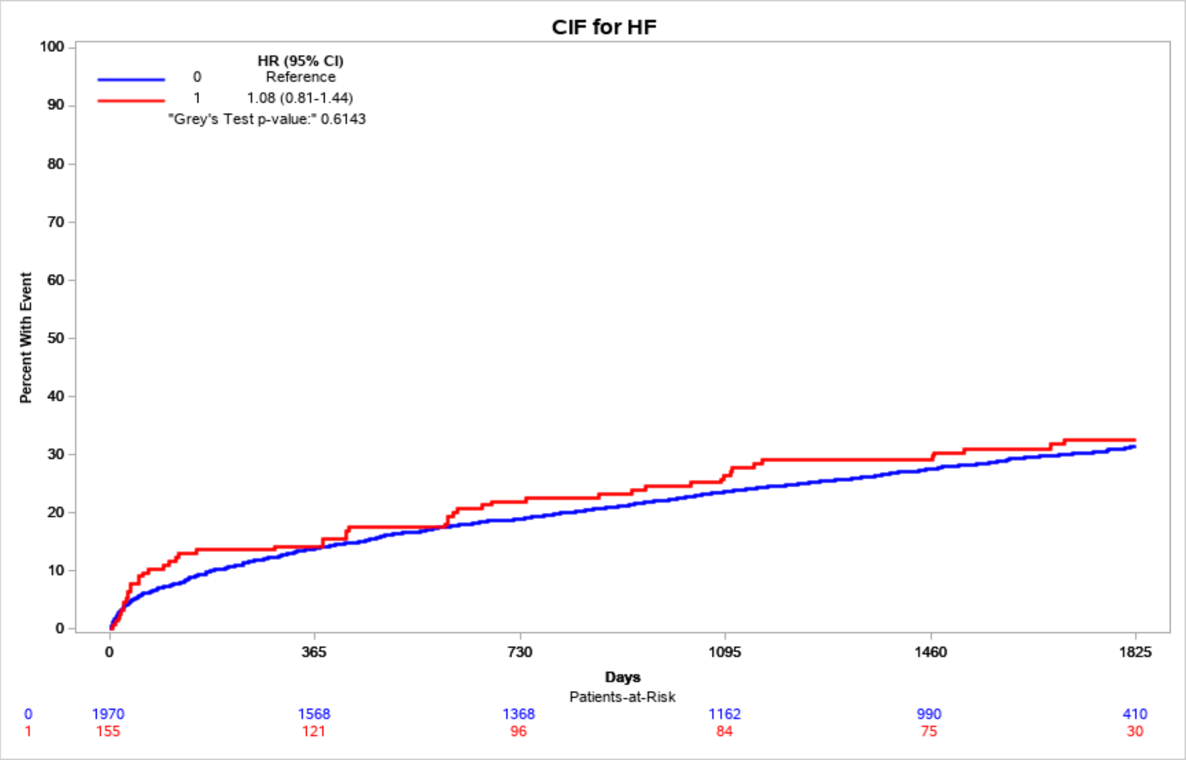

The figure shows the unadjusted survival curves from Heart Failure (HF) rehospitalization. 0 (No-significant PVL: none, trivial and mild paravalvular leak) 1 (Significant PVL: moderate and severe paravalvular leak).
